# Competing‐risks nomograms for predicting cause‐specific mortality in parotid‐gland carcinoma: A population‐based analysis

**DOI:** 10.1002/cam4.3919

**Published:** 2021-05-07

**Authors:** Fengshuo Xu, Xiaojie Feng, Fanfan Zhao, Qiao Huang, Didi Han, Chengzhuo Li, Shuai Zheng, Jun Lyu

**Affiliations:** ^1^ Department of Clinical Research The First Affiliated Hospital of Jinan University Guangzhou, Guangdong Province China; ^2^ School of Public Health Xi'an Jiaotong University Health Science Center Xi'an, Shaanxi Province China; ^3^ Center for Evidence‐Based and Translational Medicine Zhongnan Hospital of Wuhan University Wuhan, Hubei Province China; ^4^ School of Public Health Shaanxi University of Chinese Medicine Xianyang, Shaanxi Province China

**Keywords:** cause‐specific mortality, competing‐risks analysis, nomogram, parotid‐gland carcinoma, SEER

## Abstract

**Introduction:**

Parotid‐gland carcinoma (PGC) is a relatively rare tumor that comprises a group of heterogeneous histologic subtypes. We used the Surveillance, Epidemiology, and End Results (SEER) program database to apply a competing‐risks analysis to PGC patients, and then established and validated predictive nomograms for PGC.

**Methods:**

Specific screening criteria were applied to identify PGC patients and extract their clinical and other characteristics from the SEER database. We used the cumulative incidence function to estimate the cumulative incidence rates of PGC‐specific death (GCD) and other cause‐specific death (OCD), and tested for differences between groups using Gray's test. We then identified independent prognostic factors by applying the Fine–Gray proportional subdistribution hazard approach, and constructed predictive nomograms based on the results. Calibration curves and the concordance index (C‐index) were employed to validate the nomograms.

**Results:**

We finally identified 4,075 eligible PGC patients who had been added to the SEER database from 2004 to 2015. Their 1‐, 3‐, and 5‐year cumulative incidence rates of GCD were 10.1%, 21.6%, and 25.7%, respectively, while those of OCD were 2.9%, 6.6%, and 9.0%. Age, race, World Health Organization histologic risk classification, differentiation grade, American Joint Committee on Cancer (AJCC) T stage, AJCC N stage, AJCC M stage, and RS (radiotherapy and surgery status) were independent predictors of GCD, while those of OCD were age, sex, marital status, AJCC T stage, AJCC M stage, and RS. These factors were integrated for constructing predictive nomograms. The results for calibration curves and the C‐index suggested that the nomograms were well calibrated and had good discrimination ability.

**Conclusion:**

We have used the SEER database to establish—to the best of our knowledge—the first competing‐risks nomograms for predicting the 1‐, 3‐, and 5‐year cause‐specific mortality in PGC. The nomograms showed relatively good performance and can be used in clinical practice to assist clinicians in individualized treatment decision‐making.

## Graphical abstract

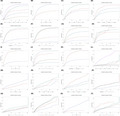

## 1 INTRODUCTION

Parotid‐gland carcinoma (PGC) comprises a relatively rare group of neoplasms that account for fewer than 3% of head and neck malignancies.^1^ Meanwhile, it also accounts for about 70% of major salivary gland carcinomas, with the other types being submandibular and sublingual gland carcinomas.^2^ Relevant studies showed that the incidence of major salivary gland carcinoma in the United States has recently increased, mainly due to PGC.^3^ Unlike the majority of head and neck malignancies being dominated by squamous cell carcinoma, PGCs are the most diverse, with at least 24 different histologic subtypes according to the World Health Organization (WHO) risk classification.^2^, ^4^, ^5^, ^6^, ^7^, ^8^, ^9^ This diversity combined with its rarity and unpredictability in long‐term outcome represent significant challenges to the overall management of PGCs.^8^, ^10^

Surgical treatment is the mainstay for PGC, including parotidectomy with or without neck dissection, and this is often accompanied by postoperative radiotherapy (PRT).^7^ Radical parotidectomy is performed in cases of facial nerve infiltration.^5^ Patients with palpable lymphadenopathy are treated with radical or modified radical neck dissection.^6^ And chemotherapy is mainly applied in the palliative setting.^11^ The treatment strategy in individual patients is determined based on evaluations of the prognosis,^5^, ^6^, ^11^, ^12^, ^13^, ^14^ and so inaccurate prognostic predictions may lead to either inadequate or excessive treatment.^15^ The commonly used prognostic tool for PGC is the American Joint Committee on Cancer (AJCC) staging, which is based on the tumor size or the extent of invasion (T), nodal involvement (N), and distant metastasis (M). However, this staging system has been applied to entire populations, and is not necessarily highly predictive in individuals;^9^, ^16^ for example, clinical experience indicates that the clinical outcome can be completely different in different patients with PGC at the same AJCC stage.^17^ These variations can be attributed to the AJCC staging not including important prognostic risk factors such as age, sex, marital status, and the degree of tumor differentiation.^18^ Nomograms have been proposed as a useful alternative to the AJCC staging to quantify the risks and estimate the prognosis of cancer patients since they can easily integrate several important individual factors into an intuitive graph with a user‐friendly interface.^9^, ^19^ Nomograms can distinguish individual prognostic differences more accurately than AJCC staging.^16^ However, we are not aware of any nomograms for predicting the prognosis of PGC patients being reported.

Most clinical oncology studies evaluate prognoses using the Kaplan–Meier method, log‐rank test, and Cox proportional‐hazards model, with all of them considering only a single endpoint.^20^ However, competing risks are common in clinical research, which refers to a situation where an individual is exposed to two or more causes of failure, and the eventual failure can be attributed to only one of them, which means that the occurrence of one type of event hinders the occurrence of any other event.^19^, ^21^ PGC is likely to be influenced by competing risks since this disease is more common in the elderly population and it has a relatively good prognosis.^4^, ^6^, ^10^, ^13^, ^22^, ^23^ This situation means that many patients may survive for longer and eventually die from non‐cancer‐related causes.^24^ If competing risks are not taken into account and the Kaplan–Meier method is used to analyze the cause‐specific mortality, other causes of death will be considered as censored.^25^, ^26^ However, this is not consistent with the important assumption that subjects experiencing censored observations should have the same survival prospects (at any specific time point) as those who continue to be followed until the outcome of interest occurs.^21^ Those who experience competing events can no longer be affected by the primary event of interest, and their inclusion in the risk set after the competing event represents “immortal” time,^27^ and so the cumulative incidence rate will be overestimated.^28^, ^29^ Although analyzing the overall mortality without distinguishing causes of death does not introduce competing‐risks bias, this approach cannot reflect the effect of factors on specific outcomes.^29^ This situation prompted Fine and Gray to propose the proportional subdistribution hazard model for analyzing cause‐specific mortality in the presence of competing risks.^30^ However, to the best of our knowledge, no competing‐risks analysis of PGC has been reported previously.

The present study aimed to evaluate and model the cumulative incidence rates of PGC‐specific death (GCD) and other cause‐specific death (OCD) for PGC patients using a competing‐risks analysis. Competing‐risks nomograms were then constructed as accurate tools individualized prediction.

## 2 METHODS

The Surveillance, Epidemiology, and End Results (SEER) database is one of the most‐representative large tumor databases in North America. This database is supported by the National Cancer Institute and collects information on cancer incidence and survival from 18 population‐based cancer registries throughout the United States, covering 28% of the total country population.^31^, ^32^ We used SEER*Stat software (version 8.3.6) to extract data on PGC patients from the following SEER subdatabase: “Incidence ‐ SEER 18 Regs Custom Data (with additional treatment fields), Nov 2018 Sub (1975–2016, varying).” The end date of follow‐up for this version of the sub‐database was December 31, 2016.

The study population comprised patients diagnosed between January 1, 2004 and December 31, 2015 who had a site code of “C07.9‐parotid gland” in ICD‐O‐3 (third edition of the International Classification of Diseases for Oncology). Patients satisfying any of the following criteria were excluded: (a) multiple primary tumors, (b) diagnosed at autopsy or by death certificate only, (c) survival time of shorter than 1 month, or (d) unknown information about race, marital status, laterality, differentiation grade, TNM stage in the sixth edition of the AJCC staging system, or cause of death (COD).

We gathered data from the SEER database on age, sex, race, marital status, laterality, histologic type, differentiation grade, AJCC T stage, AJCC N stage, AJCC M stage, chemotherapy status, radiotherapy status, surgery status, COD, and survival time. The continuous variable age was divided into five groups: <40, 40–49, 50–59, 60–69, and ≥70 years. Since there were only small numbers of American Indian/Alaska Native (AI) and Asian/Pacific Islander (API) patients, we combined them into an AI/API group. Marital status was classified into married, unmarried, and separated (including divorced, separated, and widowed). Laterality was classified into left, right, and other. Since PGCs comprise various complicated histologic types, including some exceedingly rare types, we used the following broad histologic risk classification published by the WHO in 2005 rather than specific histologic types in order to facilitate the analysis^33^: low‐risk, intermediate‐risk, and high‐risk.^23^, ^34^ Matsuda et al. found there was no difference in the overall survival (OS) between the low‐risk and intermediate‐risk WHO categories, and so we combined these into a low/intermediate‐risk category.^23^ Cases that could not be classified using this strategy were assigned to the unspecified category. The new variable RS was assigned based on a combination of the radiotherapy status and surgery status: surgery plus radiotherapy, radiotherapy alone, surgery alone, both not given. The outcome indicator COD was classified into alive, GCD, and OCD. There was a competitive relationship between GCD and OCD. The survival time was calculated from the diagnosis to death or to the end of follow‐up.

The study population was randomly divided into the training set (70%) and validation set (30%). The training set was used to perform a competing‐risks analysis and develop the study nomograms, while the validation set was used to perform external validation of the established nomograms.^35^ All of the variables are presented as frequencies and proportions except for the survival time, which is presented as median and interquartile range (IQR) values. Differences in the composition ratio of each variable between the training and validation sets were evaluated using the chi‐square test, and the Wilcoxon rank‐sum test was used to evaluate differences in the survival time between the two sets.

GCD and OCD were two competing endpoint events in the competing‐risks analysis. The cumulative incidence function (CIF) was used to estimate the 1‐, 3‐, and 5‐year cumulative incidence rates of GCD and OCD in patient groups with different characteristics.^19^ Gray's test was applied to compare differences between the different categories for each variable and thereby identify potential predictors (variables which had P value less than 0.05 in Gray's test), which corresponds to a univariate analysis.^24^ We also plotted Nelson‐Aalen curves for each potential predictor. Two main methods are currently used to analyze competing risks: the cause‐specific hazard model (CS model) and the subdistribution hazard model (SD model).^36^ In contrast to the CS model, there is a one‐to‐one relationship with the CIF for the SD model,^36^ which means that the latter is useful for direct assessments of the actual risk and is more suitable for predicting prognoses and medical decision‐making.^37^ We incorporated the potential predictors in the Fine–Gray proportional subdistribution hazard approach in order to identify independent predictors (variables which had P value less than 0.05 in Fine–Gray proportional subdistribution hazard model), which corresponds to a multivariate analysis.^24^ The subdistribution hazard ratios (sdHRs) and 95% confidence intervals (CIs) were also calculated for each predictor. Finally, we established competing‐risks nomograms based on the results from the multivariate analysis for predicting the cumulative incidence rates of GCD and OCD at 1, 3, and 5 years after a diagnosis.

The model performance was assessed based on both discrimination and calibration. The concordance index (C‐index) was used to quantify discrimination.^24^ This index reflects the concordance between predicted and observed outcomes, and ranges from 0.5 to 1.0,^9^ with a value of 0.5 indicating random chance and 1.0 indicating perfect discrimination; generally speaking, a value exceeding 0.7 is generally considered to indicate good performance.^26^ The calibration curve^16^ plots the average predicted estimate versus actual observations, and a diagonal line would indicate a perfect match between them.^25^ The calibration curve is closer to the diagonal line when the model predictions are more accurate.^9^ We also performed the same procedure for the overall survival (OS) based on the Kaplan–Meier method, log‐rank test, and Cox proportional‐hazards model.

All statistical analyses were performed using SAS software (version 9.4) as well as R software (version 4.0.0) with the following R packages: survival, cmprsk, foreign, survminer, survsim, mstate, rms, riskRegression, and pec. A two‐sided probability value of *p* < 0.05 was considered to be indicative of statistical significance.

## 3 RESULTS

We identified 4,075 eligible PGC patients who had been added to the SEER database from 2004 to 2015, and randomly allocated 2,852 of them to the training set and 1,223 to the validation set. Their baseline characteristics are presented in Table 1. The distributions of all variables except marital status were similar in the training and validation sets. Overall, 15.9%, 11.3%, 17.6%, 21.2% and 34.0% of the patients were aged <40, 40–49, 50–59, 60–69, and ≥70 years, respectively. Most of the patients were male (60.2%), white (82.2%), and married (59.5%). The lesions were almost evenly divided between left and right PGCs (49.0% vs 50.8%). The histologic risk classification was low/intermediate in 50.3% of the patients and high in 39.7% of them. Grades I (well differentiated), II (moderately differentiated), III (poorly differentiated), and IV (undifferentiated) comprised 20.2%, 34.1%, 31.6%, and 14.2% of the patients, respectively. The most common AJCC stages were T1 (31.6%), N0 (67.8%), and M0 (95.7%). Most (55.3%) of the patients received both surgery and radiotherapy, 38.7% received only surgery, 4.0% received only radiotherapy, and 2.0% had not received either surgery or radiotherapy. A large proportion of the patients (84.0%) had not received chemotherapy.

**TABLE 1:** Demographic and clinicopathological characteristics of the included patients with parotid gland carcinoma

Variables	Total (%)	Training set (%)	Validation set (%)	*p*‐value
N	4,075	2,852	1,223	
Age				
<40	647 (15.9)	464 (16.3)	183 (15.0)	0.301
40–49	462 (11.3)	334 (11.7)	128 (10.5)	
50–59	717 (17.6)	484 (17.0)	233 (19.1)	
60–69	863 (21.2)	594 (20.8)	269 (22.0)	
≥70	1,386 (34.0)	976 (34.2)	410 (33.5)	
Sex				
Male	2,454 (60.2)	1,715 (60.1)	739 (60.4)	0.889
Female	1,621 (39.8)	1,137 (39.9)	484 (39.6)	
Race				
White	3,349 (82.2)	2,348 (82.3)	1,001 (81.8)	0.273
Black	387 (9.5)	259 (9.1)	128 (10.5)	
AI/API	339 (8.3)	245 (8.6)	94 (7.7)	
Marriage				
Married	2,423 (59.5)	1,655 (58.0)	768 (62.8)	0.014
Unmarried	877 (21.5)	629 (22.1)	248 (20.3)	
Separated	775 (19.0)	568 (19.9)	207 (16.9)	
Laterality				
Left	1,998 (49.0)	1,397 (49.0)	601 (49.1)	0.893
Right	2069 (50.8)	1450 (50.8)	619 (50.6)	
Other	8 (0.2)	5 (0.2)	3 (0.2)	
Classification				
Low/Intermediate‐risk	2,048 (50.3)	1,413 (49.5)	635 (51.9)	0.361
High‐risk	1,619 (39.7)	1,152 (40.4)	467 (38.2)	
Unspecific	408 (10.0)	287 (10.1)	121 (9.9)	
Grade				
I	823 (20.2)	585 (20.5)	238 (19.5)	0.548
II	1,388 (34.1)	957 (33.6)	431 (35.2)	
III	1,287 (31.6)	896 (31.4)	391 (32.0)	
IV	577 (14.2)	414 (14.5)	163 (13.3)	
T				
T1	1,286 (31.6)	886 (31.1)	400 (32.7)	0.184
T2	1,084 (26.6)	746 (26.2)	338 (27.6)	
T3	834 (20.5)	608 (21.3)	226 (18.5)	
T4	871 (21.4)	612 (21.5)	259 (21.2)	
N				
N0	2,762 (67.8)	1,925 (67.5)	837 (68.4)	0.222
N1	548 (13.4)	401 (14.1)	147 (12.0)	
N2	738 (18.1)	505 (17.7)	233 (19.1)	
N3	27 (0.7)	21 (0.7)	6 (0.5)	
M				
M0	3,900 (95.7)	2,729 (95.7)	1,171 (95.7)	0.997
M1	175 (4.3)	123 (4.3)	52 (4.3)	
RS				
Surgery plus radiotherapy	2,253 (55.3)	1,560 (54.7)	693 (56.7)	0.672
Radiotherapy alone	164 (4.0)	118 (4.1)	46 (3.8)	
Surgery alone	1,578 (38.7)	1,116 (39.1)	462 (37.8)	
Both not given	80 (2.0)	58 (2.0)	22 (1.8)	
Chemotherapy				
No/Unknown	3,422 (84.0)	2,395 (84.0)	1,027 (84.0)	1.000
Yes	653 (16.0)	457 (16.0)	196 (16.0)	
COD				
Alive	2,683 (65.8)	1,846 (64.7)	837 (68.4)	0.071
GCD	974 (23.9)	702 (24.6)	272 (22.2)	
OCD	418 (10.3)	304 (10.7)	114 (9.3)	
Survival times				
Median (IQR)	43 (19–84)	42 (19–85)	44 (21–84)	0.269

Abbreviations: AI, American Indian/Alaska Native; API, Asian/Pacific Islander; COD, cause of death; GCD, PGC‐specific death; OCD, other cause‐specific death; PGC, parotid gland carcinoma; RS, radiotherapy and surgery status.

**FIGURE 1:**
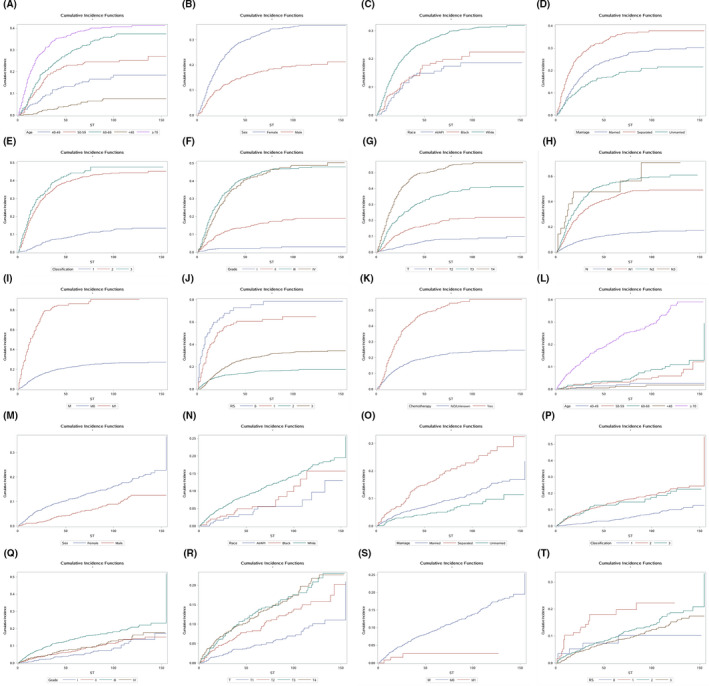
Nelson‐Aalen curves for each characteristic. (A)‐ (K) for GCD, (L)‐ (T) for OCD. Abbreviations: AI, American Indian/Alaska Native; API, Asian/Pacific Islander; RS, radiotherapy and surgery status; PGC, parotid gland carcinoma; GCD, PGC‐specific death; OCD, other cause‐specific death

The median follow‐up was 43 months (IQR=19–84 months), during which 1,392 (34.2%) patients died: 974 (23.9%) were GCD and 418 (10.3%) were OCD. Table 2 presents the 1‐, 3‐, and 5‐year estimates of the cumulative incidence rates of GCD and OCD according to different characteristics and the results of Gray's test. The 1‐, 3‐, and 5‐ year incidence rates were 10.1%, 21.6%, and 25.7%, respectively, for GCD, and 2.9%, 6.6%, and 9.0% for OCD. The univariate analyses showed that all variables except laterality were potential predictors of GCD, while nine variables (which excluded laterality, AJCC N stage, and chemotherapy status) were potentially correlated with OCD. Figure 1 shows the corresponding Nelson‐Aalen curves for the potential predictors.

**TABLE 2:** Cumulative incidence rates of cause‐specific death and Gray's test in the training set

Variables	GCD (%)				OCD (%)			
	1‐Year	3‐Year	5‐Year	*p*‐value	1‐Year	3‐Year	5‐Year	*p*‐value
Total	10.1	21.6	25.7		2.9	6.6	9.0	
Age								
<40	0.4	2.6	4.8	<0.001	0.0	0.4	0.8	<0.001
40–49	4.5	11.6	14.3		0.3	0.6	1.4	
50–59	7.5	19.4	22.9		1.0	2.2	3.1	
60–69	8.2	23.2	30.2		1.7	2.8	3.5	
≥70	19.0	34.1	38.2		6.8	16.0	21.7	
Sex								
Male	12.4	26.8	31.5	<0.001	4.1	8.8	11.5	<0.001
Female	6.7	13.7	16.9		1.2	3.2	5.3	
Race								
White	11.1	23.3	27.5	<0.001	3.2	7.3	9.8	0.006
Black	7.0	13.5	18.3		2.0	3.3	5.6	
AI/API	4.2	13.1	15.7		0.8	2.7	4.8	
Marriage								
Married	8.5	20.8	25.4	<0.001	2.7	5.8	8.0	<0.001
Unmarried	7.3	15.0	17.7		1.8	3.3	4.8	
Separated	17.8	31.0	35.2		4.8	12.6	16.7	
Laterality								
Left	11.0	22.0	26.6	0.362	3.3	6.5	9.1	0.734
Right	9.2	21.0	24.7		2.6	6.7	8.9	
Other	20.0	46.7	46.7		0.0	0.0	0.0	
Classification								
Low/intermediate‐risk	1.7	7.1	9.3	<0.001	0.8	2.2	3.7	<0.001
High‐risk	17.7	34.8	40.9		4.8	10.6	14.4	
Unspecific	21.0	39.1	44.3		5.6	11.8	12.8	
Grade								
I	1.4	2.1	2.1	<0.001	0.7	2.7	4.8	<0.001
II	6.2	12.9	15.4		2.2	5.0	7.1	
III	18.2	37.0	43.7		5.2	11.1	14.3	
IV	13.9	35.2	42.4		2.7	5.8	7.8	
T								
T1	1.7	5.1	7.9	<0.001	1.4	3.5	4.6	<0.001
T2	6.1	14.9	17.8		3.0	6.1	8.3	
T3	14.2	28.0	35.2		3.8	9.2	13.2	
T4	23.0	46.8	51.1		4.1	9.0	12.0	
N								
N0	5.2	11.1	14.0	<0.001	2.7	5.9	8.3	0.354
N1	16.6	36.9	43.4		2.5	8.5	12.0	
N2	22.5	48.2	55.0		4.2	7.8	9.6	
N3	33.3	47.6	47.6		0.0	0.0	0.0	
M								
M0	8.4	18.8	23.0	<0.001	3.0	6.8	9.3	0.005
M1	47.4	82.8	86.3		0.8	2.6	2.6	
RS								
Surgery plus radiotherapy	8.0	23.1	28.4	<0.001	2.1	5.7	8.1	0.004
Radiotherapy alone	37.0	58.1	60.7		10.3	18.0	18.0	
Surgery alone	8.1	13.1	15.6		3.2	6.6	9.4	
Both not given	50.4	70.4	75.7		3.4	7.4	7.4	
Chemotherapy								
No/Unknown	8.5	17.6	21.1	<0.001	2.9	6.9	9.4	0.075
Yes	18.3	42.3	49.8		2.9	4.8	7.2	

Abbreviations: AI, American Indian/Alaska Native; API, Asian/Pacific Islander; GCD, PGC‐specific death; OCD, other cause‐specific death; PGC, parotid gland carcinoma; RS, radiotherapy and surgery status.

The results of the multivariable analysis presented in Table 3 indicate that the independent predictors of GCD were age, race, WHO histologic risk classification, differentiation grade, AJCC T stage, AJCC N stage, AJCC M stage, and RS. However, the prognostic value of sex, marital status, and chemotherapy status disappeared after adjusting the potential predictors identified in the univariate analyses. The risk of GCD was higher in older patients. The cumulative incidence rate of GCD was lower in black than white patients (sdHR = 0.671, 95% CI = 0.467–0.964), while it did not differ between AI/API and white patients. The WHO histologic risk classification was a significant independent predictor for GCD, with an sdHR of 1.283 (95% CI = 1.006–1.637) for patients with high‐risk histologic types compared with patients with low/intermediate‐risk histologic types. The cumulative incidence of GCD was also higher for an advanced differentiation grade, advanced AJCC T stage, advanced AJCC N stage, and distant metastases (AJCC M stage). The risk of GCD did not differ significantly between patients who received both surgery and radiotherapy and those who received only surgery, while it was higher in patients who received only radiotherapy (vs. surgery plus radiotherapy: sdHR = 1.619, 95% CI = 1.184–2.214), and highest in those who had not received either treatment (vs. surgery plus radiotherapy: sdHR = 3.580, 95% CI = 2.310–5.547). The independent predictors of OCD were age, sex, marital status, AJCC T stage, AJCC M stage, and RS, but not race, WHO histologic risk classification, or differentiation grade.

**TABLE 3:** Fine and Gray's proportional subdistribution hazard analysis for cause‐specific death in the training set

Variables	GCD				OCD			
	Coefficient	SdHR	95% CI	*p*‐value	Coefficient	SdHR	95% CI	*p*‐value
Age								
<40	Reference				Reference			
40–49	0.507	1.661	1.045–2.640	0.032	0.618	1.855	0.556–6.185	0.315
50–59	0.772	2.165	1.403–3.340	0.001	1.412	4.106	1.507–11.190	0.006
60–69	0.856	2.354	1.538–3.601	<0.001	1.719	5.580	2.114–14.727	0.001
≥70	1.137	3.117	2.049–4.742	<0.001	3.124	22.746	8.924–57.974	<0.001
Sex								
Male	–	–	–	–	Reference			
Female	–	–	–	–	−0.496	0.609	0.459–0.808	0.001
Race								
White	Reference				–	–	–	–
Black	−0.399	0.671	0.467–0.964	0.031	–	–	–	–
AI/API	−0.212	0.809	0.584–1.121	0.203	–	–	–	–
Marriage								
Married	–	–	–	–	Reference			
Unmarried	–	–	–	–	0.181	1.198	0.808–1.776	0.368
Separated	–	–	–	–	0.377	1.458	1.125–1.890	0.004
Classification								
Low/intermediate‐risk	Reference				–	–	–	–
High‐risk	0.249	1.283	1.006–1.637	0.045	–	–	–	–
Unspecific	0.176	1.193	0.878–1.621	0.260	–	–	–	–
Grade								
I	Reference				–	–	–	–
II	1.388	4.005	2.298–6.982	<0.001	–	–	–	–
III	1.715	5.555	3.084–10.007	<0.001	–	–	–	–
IV	1.821	6.178	3.413–11.181	<0.001	–	–	–	–
T								
T1	Reference				Reference			
T2	0.537	1.712	1.261–2.324	0.001	0.411	1.509	1.056–2.157	0.024
T3	0.963	2.619	1.944–3.528	<0.001	0.554	1.740	1.204–2.516	0.003
T4	1.277	3.585	2.673–4.808	<0.001	0.432	1.540	1.052–2.254	0.027
N								
N0	Reference				–	–	–	–
N1	0.564	1.757	1.424–2.168	<0.001	–	–	–	–
N2	0.661	1.936	1.585–2.366	<0.001	–	–	–	–
N3	0.921	2.513	1.323–4.773	0.005	–	–	–	–
M								
M0	Reference				Reference			
M1	1.026	2.791	2.110–3.692	<0.001	−2.045	0.129	0.041–0.409	0.001
RS								
Surgery plus radiotherapy	Reference				Reference			
Radiotherapy alone	0.482	1.619	1.184–2.214	0.003	0.430	1.538	0.952–2.483	0.079
Surgery alone	0.004	1.004	0.823–1.224	0.970	0.447	1.564	1.219–2.006	<0.001
Both not given	1.275	3.580	2.310–5.547	<0.001	−0.470	0.625	0.251–1.554	0.312

Abbreviations: AI, American Indian/Alaska Native; API, Asian/Pacific Islander; GCD, PGC‐specific death; OCD, other cause‐specific death; PGC, parotid gland carcinoma; RS, radiotherapy and surgery status.

**FIGURE 2:**
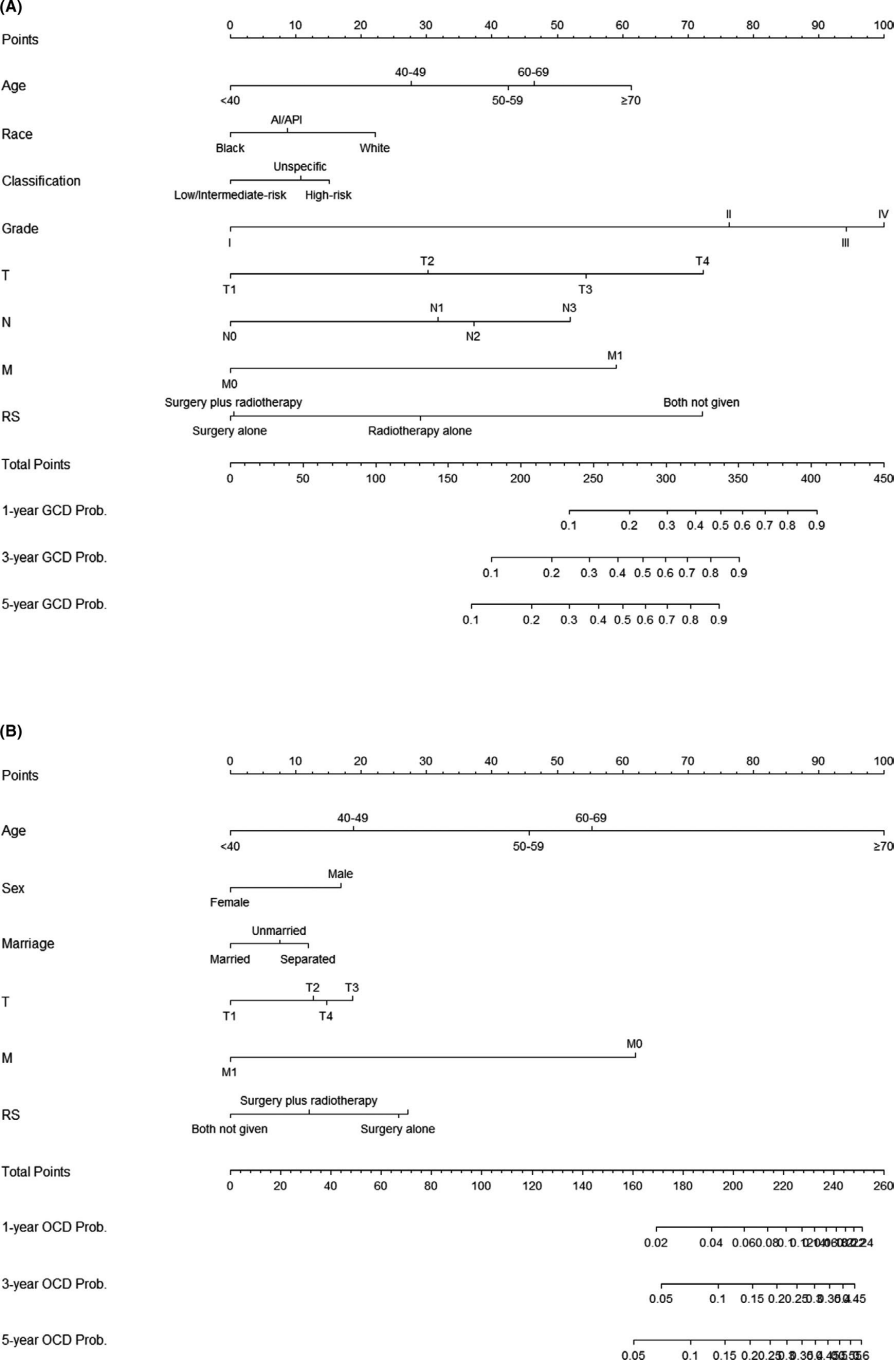
Competing‐risk nomograms for predicting 1‐, 3‐ and 5‐years cumulative incidence rates of GCD and OCD in patients with parotid gland carcinoma. (A) for GCD; (B) for OCD. Abbreviations: AI, American Indian/Alaska Native; API, Asian/Pacific Islander; RS, radiotherapy and surgery status; PGC, parotid gland carcinoma; GCD, PGC‐specific death; OCD, other cause‐specific death

**FIGURE 3:**
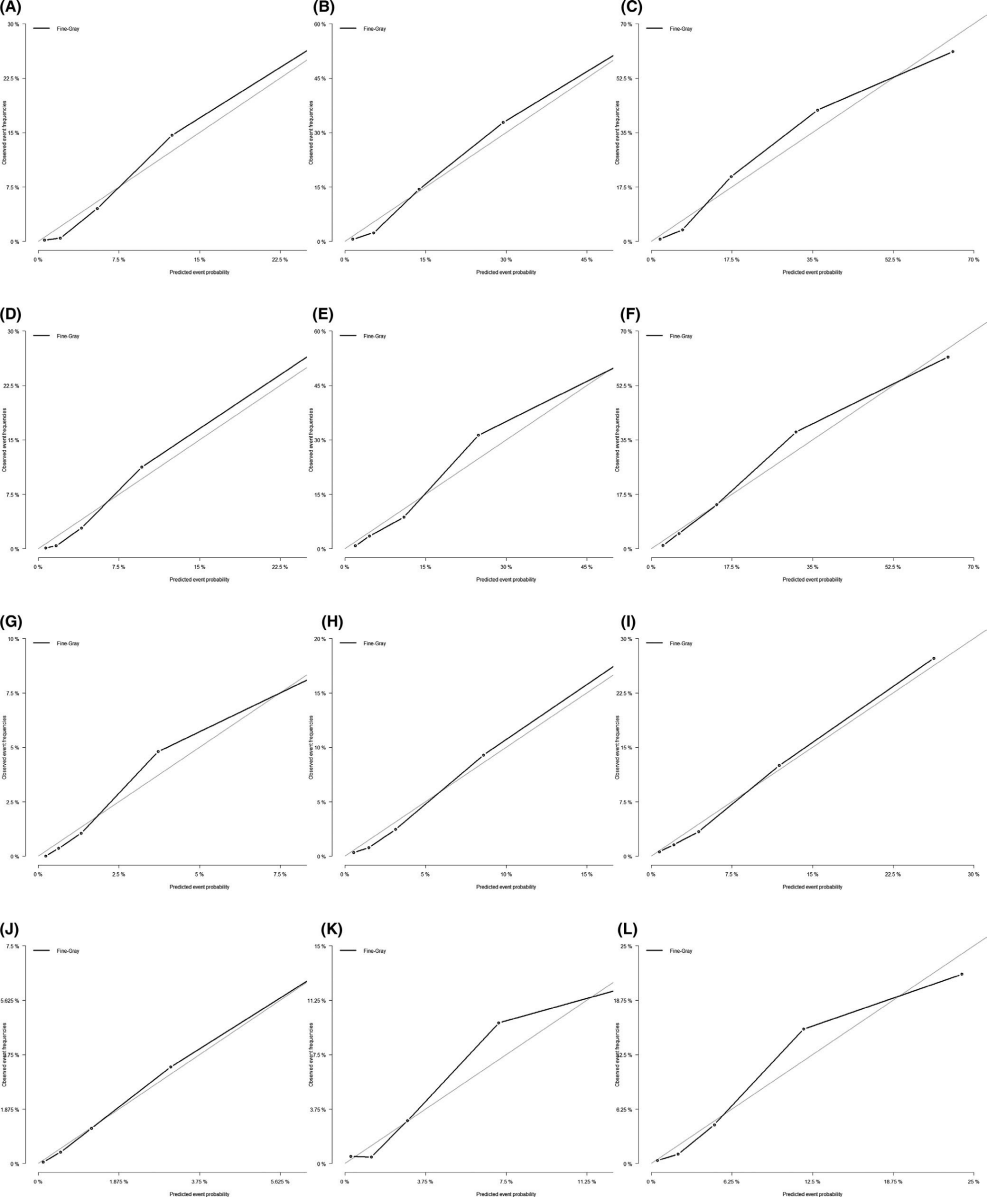
Calibration curves for 1‐, 3‐ and 5‐years prediction. (A)‐ (F) for GCD; (A)1‐year, (B) 3‐year, (C)5‐year for internal validation; (D) 1‐year, (E) 3‐year, (F)5‐year for external validation. (G)‐ (L) for OCD; (G) 1‐year, (H) 3‐year, (I) 5‐year for internal validation; (J) 1‐year, (K) 3‐year, (L) 5‐year for external validation

The nomograms constructed based on the results obtained in the Fine–Gray proportional subdistribution hazard analysis are shown in Figure 2. These nomograms can be used to predict the cumulative incidence rates of GCD and OCD at 1, 3, and 5 years after a diagnosis, by summating the scores for an individual patient.^38^ Both nomograms exhibited good discrimination ability, as indicated by their relatively high C‐indexes at 1, 3, and 5 years in the training set (0.837, 0.829, and 0.818, respectively, for GCD, and 0.824, 0.823, and 0.818, respectively, for OCD) and in the validation set (0.858, 0.833, 0.828, 0.818, 0.792, and 0.794, respectively). The calibration curves shown in Figure 3 are all close to the diagonal line, which indicates that the nomograms were well calibrated.^25^

Table S1 lists the 1‐, 3‐, and 5‐year OS rates according to different characteristics estimated using the Kaplan–Meier method, univariate analyses with the log‐rank test, and multivariate analysis with the Cox proportional‐hazards model. Figure S1 presents the corresponding OS curves for different variables. The 1‐, 3‐, and 5‐year OS rates were 87.0%, 71.8%, and 65.3%, respectively, and the independent predictors for OS were age, sex, marital status, WHO histologic risk classification, differentiation grade, AJCC T stage, AJCC N stage, AJCC M stage, and RS. Figure S2 shows the nomogram that we constructed based on the results of the Cox proportional‐hazards analysis for predicting the OS rates of PGC patients at 1, 3, and 5 years after a diagnosis. The calibration curves in Figure S3 suggest that this nomogram was well calibrated, while the relatively high C‐indexes at 1, 3, and 5 years in the training set (0.844, 0.829, and 0.821, respectively) and in the validation set (0.855, 0.832, and 0.826, respectively) indicated the good discrimination ability of the nomogram.

## 4 DISCUSSION

It is well known that the ability to accurately predict the prognosis of PGC is critical to the development of treatment strategies. However, the overall rarity of PGC has resulted in determination of the significant prognostic factors being largely dependent on single institutional series involving small samples.^22^ Moreover, the lack of representativeness of the samples makes it difficult to extrapolate the conclusions of such studies. The present study performed analyses based on the SEER database, which is derived from 18 population‐based cancer registries and represents approximately 28% of the total population of the United States.^26^ Each SEER registry collects data on patient demographics, primary tumor site and histology, stage of cancer at the time of diagnosis, the first course of treatment, and the survival time during follow‐up.^39^ The high‐quality, multicenter, large‐sample clinical data provided by the SEER program facilitates a broad and reliable approach to the study of tumors, especially rare tumors.^40^, ^41^ We identified 4,075 eligible patients from the SEER database, which represents a relatively large sample for a study involving PGC, which greatly improves the ability to extrapolate the present results.

By the end of follow‐up, 1,392 patients (34.2%) had died: 974 (23.9%) were PGC and 418 (10.3%) were OCD. There is a strong competitive relationship between GCD and OCD. If the traditional Kaplan–Meier method is used to estimate the cumulative incidence rate of a cause‐specific death, the corresponding competing event will be considered as censored, and so the rate will be overestimated. In comparison, utilizing the CIF that takes competing risks into account may provide estimates that are less biased. In this study, the cumulative incidence rates at 1, 3, and 5 years after a diagnosis were 10.1%, 21.6%, and 25.7%, respectively, for GCD, and 2.9%, 6.6%, and 9.0% for OCD. These findings demonstrate that for a longer follow‐up there will be more patients who die from other causes, and hence that OCD has a strong competitive effect on GCD during a long‐term follow‐up.

Since absolute risks in the real world where competing events can occur are more important for prognosis predictions and medical decision‐making, the SD model is more suitable than the CS model.^28^ We observed that age remained a predictor for GCD and OCD after adjustment by the SD model, with older patients more likely to have higher cumulative incidence rates of GCD and OCD. Many other studies have also supported the prognostic value of age. Huang et al. found that the overall mortality was 23.614 times (95% CI = 2.606–213.958) higher for PGC patients aged ≥50 years that for those aged <50 years.^12^ Erovic et al. similarly found that patients younger than 60 years had a better disease‐free survival than older patients.^10^ We surmise that immunologic or other age‐associated factors may have played a role in the aforementioned findings. We found that the cumulative incidence rates of OCD and GCD were 22.74 times higher (95% CI = 8.924–57.974) and 3.117 times higher (95% CI = 2.049–4.742), respectively, in patients aged ≥70 years than in those <40 years old. This indicates that age had a greater influence on OCD, and that more elderly patients (especially those aged ≥70 years) died from causes other than PGC. This intense competition of OCD on GCD may be due to the higher likelihood of comorbidities among the older population. This further suggests that treating PGC alone in elderly patients will not achieve large survival benefits, and so more attention should be paid to the treatment of comorbidities.^29^

Previous studies have suggested that black patients tend to have a worse prognosis than white patients due to their worse overall economic status and access to health care. However, we obtained the opposite result, with the cumulative incidence of GCD being lower in black patients than white patients (sdHR = 0.671, 95% CI = 0.467–0.964). We consider that our results are reliable since they are based on multicenter, large‐sample data and a competing‐risks analysis. It could be that there are certain factors that accelerate the development of cancer in white patients. But it is worth noting that the number of White patients registered in SEER were significantly higher than black patients, which may have biased our study. Therefore, the influence of race on the prognosis of PGC patients still needs to be explored in future prospective studies. Stodulski et al. found that the OS tended to be poor in males, ^1^ and our study similarly found that female patients had a lower risk of OCD than did male patients (sdHR = 0.609, 95% CI = 0.459–0.808). This might be due to males being more likely to be exposed to various risks. Few previous studies of PGC have included the marital status as a potential predictor in their analyses. The present study found that the risk of OCD did not differ between married and unmarried patients, whereas the risk was lower in married patients than in separated ones (including divorced, separated, and widowed patients: sdHR = 1.458, 95% CI = 1.125–1.890). This survival benefit might be due to receiving greater social and financial support from the family.^31^

Our study also found that the WHO histologic risk classification had important prognostic value, with the risk of GCD in the high‐risk group being 1.283 times higher (95% CI = 1.006–1.637) than that in the low/intermediate‐risk group. The differentiation grade reflects the intrinsic qualities of a tumor. A systematic review implicated that survival is worse in high‐grade PGC than in low‐grade PGC.^6^ We similarly found that a higher differentiation grade was associated with a higher risk of GCD. The AJCC staging system is a commonly used tool to predict the prognosis of PGC, and this study found that the AJCC T, N, and M stages are very important prognostic factors. An interesting finding is that the OCD of M0 stage is higher than that of M1 stage. This may be because the effect of M stage on GCD is so great that patients with M1 stage are more likely to die from PGC, which competitively leads to a decrease in the number of M1 patients who die from other causes.

The prognostic value of PRT has been explored in several studies, and it remains controversial, with some authors believing that PRT can significantly improve the prognosis of PGC patients,^23^ others considering that PGC patients cannot benefit from PRT,^6^, ^22^ and some even thinking that PRT will impair survival due to its adverse effects.^11^, ^42^ The present study found no significant difference in the GCD risk between patients who received both surgery and radiotherapy and those who received only surgery. We attributed this to PRT often being used in patients with a more‐advanced disease status. However, the positive effects of radiotherapy cannot be denied, since patients who received only radiotherapy still had a lower risk of GCD than those who did not receive either treatment (only radiotherapy vs surgery plus radiotherapy: sdHR = 1.619, 95% CI = 1.184–2.214; neither radiotherapy nor surgery vs surgery plus radiotherapy: sdHR = 3.580, 95% CI = 2.310–5.547). Almost all of the few studies of the effects of chemotherapy concluded that this treatment does not improve the survival of PGC patients,^12^, ^13^, ^14^, ^23^, ^34^ and we obtained the same result. However, Andry et al. suggested that chemotherapy was effective in preventing the recurrence of PGC and can increase the disease‐free survival rate of patients.^42^

Based on the results obtained in the present Fine–Gray subdistribution hazard analysis, we constructed nomograms to predict the cumulative incidence rates of GCD and OCD at 1, 3, and 5 years after a diagnosis of PGC. These nomograms include a wide range of clinical risk factors that can readily be collected from historical medical records. The well‐fitted calibration curves and relatively high C‐indexes indicate that they performed well. A major advantage of nomograms is that they provide individual patients with tailored outcomes and allow risk assessments to be performed based on individual factors, which contrasts with obtaining a relative risk applicable to a particular group or condition.^16^ Numerous clinical oncology studies have shown that nomograms can predict prognoses more accurately than the use of the AJCC staging system alone.^18^, ^19^, ^20^, ^24^, ^25^, ^26^, ^38^ Each nomogram is also very easy to use: the factors for each predictor are given a points score and its corresponding line is then marked according to the set scale, and the total score for the nomogram is then obtained by summing the scores for all of the predictors, and this is subsequently converted into the cumulative incidence rates of 1‐, 3‐, and 5‐year GCD or OCD for PGC patients.^17^ Clinicians can use these nomograms to obtain more‐accurate prognostic information about individual PGC patients, resulting in better patient counseling and personalized treatment decision‐making. The nomograms can also be used when designing clinical studies.^26^

We further analyzed the OS of PGC patients and established a predictive nomogram. The predictors for OS were the combination of those for GCD and OCD. In the presence of competing risks, the traditional survival analysis based on the Kaplan–Meier method can be used to analyze the composite endpoint, but competing‐risks analysis may provide further insights into the effect of prognostic factors on specific endpoints.^28^

The strengths of our study are its use of high‐quality, multicenter, large‐sample data and the application of competing‐risks analysis, which increased the reliability of our results. However, we have to admit that our study was also subject to some limitations. First, many cases were not included in the study due to missing information, which may have led to selection bias. Second, there could be many significant prognostic factors that are not documented in the SEER database, such as surgical margins, perineural invasion, and facial nerve palsy. A nomogram generally cannot include all prognostic factors, and so the value predicted from using it only represents a reference for clinicians when making decisions, rather than providing an absolute accurate prognosis. Third, recurrence is also an important outcome, but this is not provided in the SEER follow‐up information. We were therefore unable to determine individualized estimates of the risk of recurrence. Fourth, our nomograms are limited by the retrospective nature of the data collection and have only been validated using the SEER database. Therefore, the nomograms must be further validated externally using prospective cohorts before they are applied clinically.

## 5 CONCLUSION

We have used the SEER database to evaluate and model the cumulative incidence rates of cause‐specific death in PGC patients in a competing‐risks analysis. The results obtained have been used to construct—to the best of our knowledge—the first competing‐risks nomograms for predicting the 1‐, 3‐, and 5‐year incidence rates of mortality in PGC. The ease of use and relatively good performance of the nomograms indicate that they can be used in clinical practice to assist clinicians making decisions about individualized treatments. However, further external validation is still needed.

## ETHICAL APPROVAL AND INFORMED CONSENT

All procedures performed in the present study were in accordance with the principles outlined in the 1964 Helsinki Declaration and its later amendments. Institutional review board approval and informed consent were not required in current study because SEER research data is publicly available and all patient data are de‐identified.

## CONFLICT OF INTEREST

The authors report no conflicts of interest in this work.

## AUTHOR CONTRIBUTION

FX, XF and FZ analyzed the data and wrote the paper; QH and DH collected the data; CL and SZ checked the integrity of the data and the accuracy of the data analysis; FX and JL designed the study and revised the paper. All authors read and approved the final manuscript.

## Supporting information

Fig S1

Fig S2

Fig S3

Table S1

## Data Availability

The data sets generated and/or analyzed during the current study are available in the SEER database (https://seer.cancer.gov/).
