# Supplementary figures and images for: Competing‐risks nomograms for predicting cause‐specific mortality in parotid‐gland carcinoma: A population‐based analysis
Source: Cancer Med. 2021 May 7;10(11):3756–69. doi: 10.1002/cam4.3919 (PMC8178487; doi:10.1002/cam4.3919)

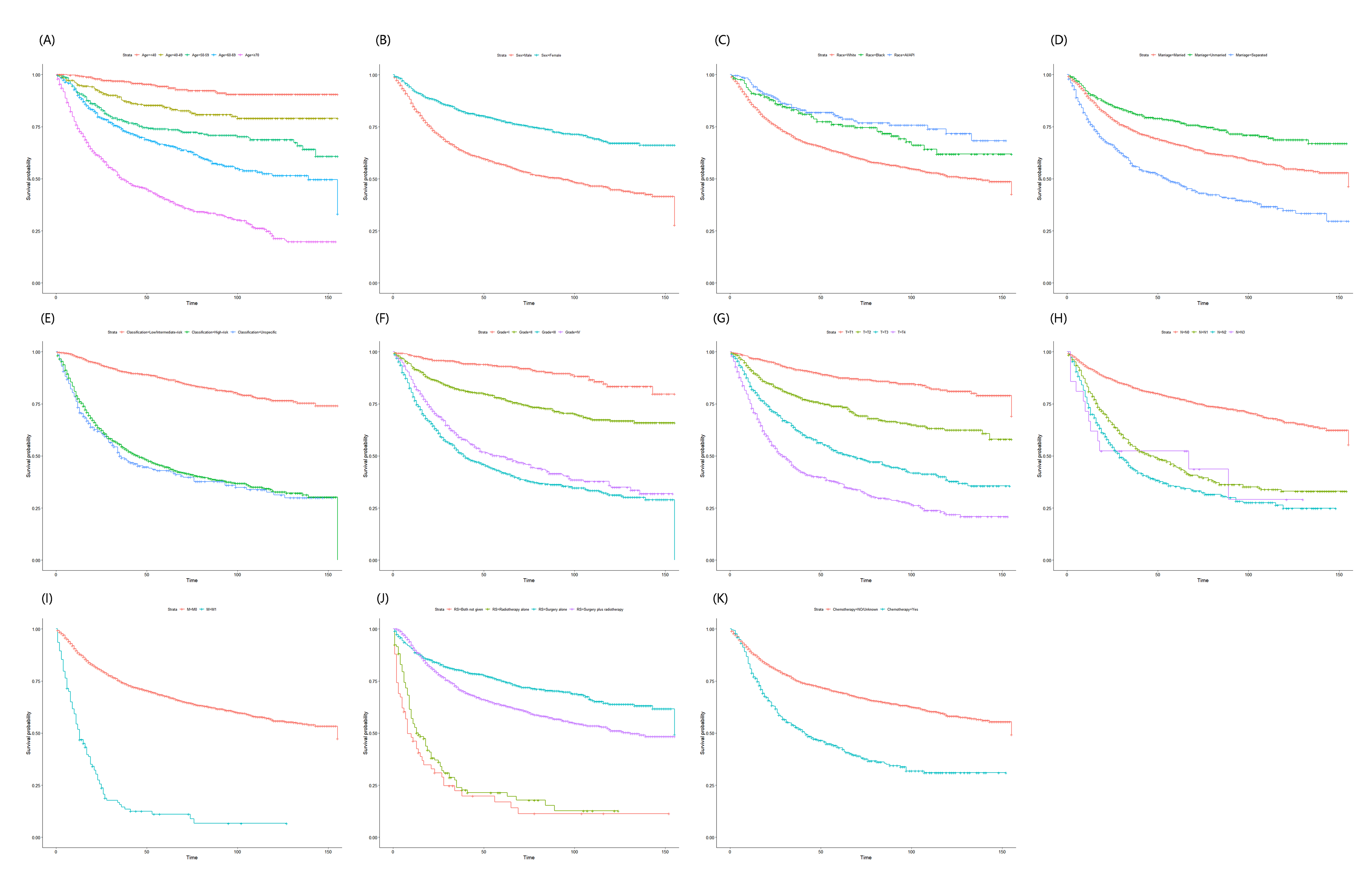

Supplement: Supplementary file 1 — Fig S1 [file CAM4-10-3756-s004.tif]

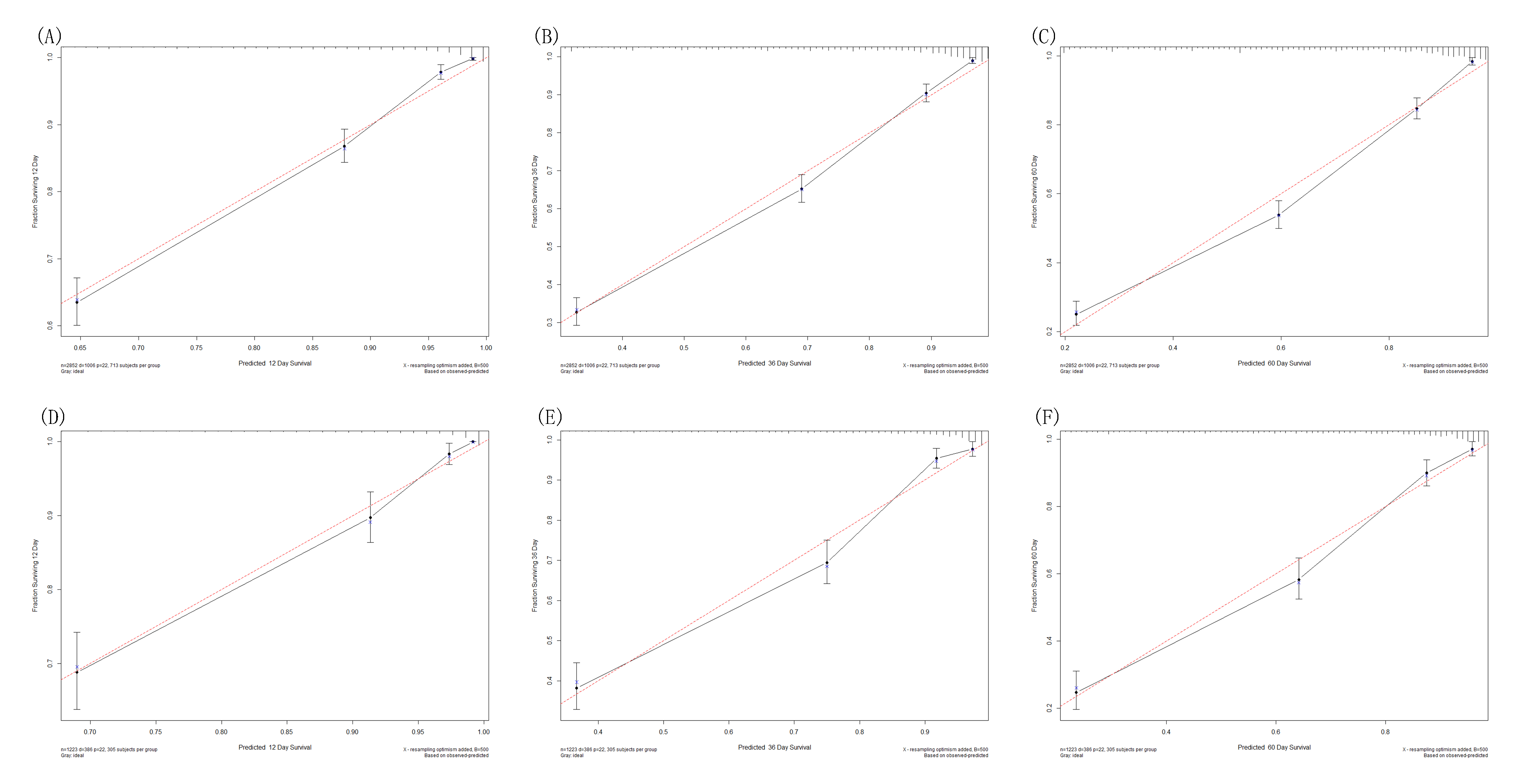

Supplement: Supplementary file 3 — Fig S3 [file CAM4-10-3756-s003.tif]
