# Supplementary material for: Competing‐risks nomograms for predicting cause‐specific mortality in parotid‐gland carcinoma: A population‐based analysis
Source: Cancer Med. 2021 May 7;10(11):3756–69. doi: 10.1002/cam4.3919 (PMC8178487; doi:10.1002/cam4.3919)
Supplement: Supplementary file 4 — Table S1 [file CAM4-10-3756-s001.docx]

Table S1. Univariate and Multivariate analysis for overall survival in the training set.

| **Variables** | **OS (%)** | | | **Univariate Analysis** | **Multivariate Analysis** | | | |
| --- | --- | --- | --- | --- | --- | --- | --- | --- |
|  | **1-Year** | **3-Year** | **5-Year** | **P-value** | **Coefficient** | **HR** | **95%CI** | **P-value** |
| **Total** | 87.0 | 71.8 | 65.3 |  |  |  |  |  |
| **Age** |  |  |  | ＜0.001 |  |  |  |  |
| <40 | 99.6 | 96.9 | 94.4 |  | Reference |  |  |  |
| 40-49 | 95.2 | 87.7 | 84.3 |  | 0.691 | 1.996 | 1.260-3.162 | 0.003 |
| 50-59 | 91.4 | 78.4 | 74.0 |  | 1.034 | 2.811 | 1.850-4.272 | ＜0.001 |
| 60-69 | 90.1 | 74.0 | 66.3 |  | 1.160 | 3.190 | 2.124-4.790 | ＜0.001 |
| ≥70 | 74.2 | 49.9 | 40.2 |  | 1.923 | 6.840 | 4.585-10.204 | ＜0.001 |
| **Sex** |  |  |  | ＜0.001 |  |  |  |  |
| Male | 83.6 | 64.4 | 57.0 |  | Reference |  |  |  |
| Female | 92.2 | 83.1 | 77.8 |  | -0.245 | 0.783 | 0.673-0.911 | 0.002 |
| **Race** |  |  |  | ＜0.001 |  |  |  |  |
| White | 85.7 | 69.4 | 62.7 |  | - | - | - | - |
| Black | 91.0 | 83.2 | 76.1 |  | - | - | - | - |
| AI/API | 95.0 | 84.1 | 79.5 |  | - | - | - | - |
| **Marriage** |  |  |  | ＜0.001 |  |  |  |  |
| Married | 88.8 | 73.4 | 66.6 |  | Reference |  |  |  |
| Unmarried | 91.0 | 81.6 | 77.6 |  | 0.162 | 1.176 | 0.972-1.422 | 0.096 |
| Separated | 77.3 | 56.4 | 48.1 |  | 0.265 | 1.304 | 1.124-1.512 | 0.001 |
| **Laterality** |  |  |  | 0.05 |  |  |  |  |
| Left | 85.7 | 71.5 | 64.3 |  | - | - | - | - |
| Right | 88.2 | 72.3 | 66.4 |  | - | - | - | - |
| Other | 80.0 | 53.3 | 53.3 |  | - | - | - | - |
| **Classification** |  |  |  | ＜0.001 |  |  |  |  |
| Low/Intermediate-risk | 97.5 | 90.7 | 87.0 |  | Reference |  |  |  |
| High-risk | 77.5 | 54.6 | 44.7 |  | 0.394 | 1.483 | 1.218-1.806 | ＜0.001 |
| Unspecific | 73.3 | 49.0 | 43.0 |  | 0.328 | 1.388 | 1.082-1.781 | 0.010 |
| **Grade** |  |  |  | ＜0.001 |  |  |  |  |
| I | 97.9 | 95.2 | 93.1 |  | Reference |  |  |  |
| II | 91.6 | 82.1 | 77.5 |  | 0.605 | 1.831 | 1.340-2.503 | ＜0.001 |
| III | 76.6 | 51.8 | 42.0 |  | 0.835 | 2.306 | 1.669-3.186 | ＜0.001 |
| IV | 83.4 | 59.0 | 49.8 |  | 0.852 | 2.345 | 1.674-3.283 | ＜0.001 |
| **T** |  |  |  | ＜0.001 |  |  |  |  |
| T1 | 96.9 | 91.4 | 87.5 |  | Reference |  |  |  |
| T2 | 90.9 | 79.0 | 73.9 |  | 0.579 | 1.785 | 1.411-2.257 | ＜0.001 |
| T3 | 82.0 | 62.7 | 51.5 |  | 0.977 | 2.656 | 2.115-3.337 | ＜0.001 |
| T4 | 72.9 | 44.2 | 36.9 |  | 1.219 | 3.385 | 2.704-4.236 | ＜0.001 |
| **N** |  |  |  | ＜0.001 |  |  |  |  |
| N0 | 92.1 | 83.0 | 77.7 |  | Reference |  |  |  |
| N1 | 80.9 | 54.5 | 44.6 |  | 0.311 | 1.364 | 1.153-1.614 | 0.000 |
| N2 | 73.3 | 44.0 | 35.4 |  | 0.508 | 1.662 | 1.415-1.953 | ＜0.001 |
| N3 | 66.7 | 52.4 | 52.4 |  | 0.270 | 1.310 | 0.734-2.339 | 0.361 |
| **M** |  |  |  | ＜0.001 |  |  |  |  |
| M0 | 88.6 | 74.4 | 67.7 |  | Reference |  |  |  |
| M1 | 51.8 | 14.6 | 11.1 |  | 0.799 | 2.224 | 1.758-2.814 | ＜0.001 |
| **RS** |  |  |  | ＜0.001 |  |  |  |  |
| Surgery plus radiotherapy | 89.8 | 71.2 | 63.4 |  | Reference |  |  |  |
| Radiotherapy alone | 52.7 | 23.9 | 21.4 |  | 0.725 | 2.064 | 1.616-2.637 | ＜0.001 |
| Surgery alone | 88.8 | 80.3 | 75.0 |  | 0.264 | 1.302 | 1.122-1.511 | ＜0.001 |
| Both not given | 46.1 | 22.3 | 17.0 |  | 1.316 | 3.729 | 2.683-5.185 | ＜0.001 |
| **Chemotherapy** |  |  |  | ＜0.001 |  |  |  |  |
| NO/Unknown | 88.6 | 75.5 | 69.6 |  | - | - | - | - |
| Yes | 78.8 | 52.9 | 43.0 |  | - | - | - | - |

Abbreviations: AI, American Indian/Alaska Native; API, Asian/Pacific Islander; OS, overall survival.
